# Effects of Stocking Density on Milk Fatty Acids Composition and Oxidative Stability of Mid- and Late-Lactating Dairy Cows

**DOI:** 10.3390/ani8090148

**Published:** 2018-08-21

**Authors:** Shixin Lin, Jianxin Liu, Kaiying Wang, Diming Wang

**Affiliations:** 1 Institute of Dairy Science, MoE Key Laboratory of Molecular Animal Nutrition, College of Animal Sciences, Zhejiang University, Hangzhou 310058, China; linshixin@zju.edu.cn (S.L.); liujx@zju.edu.cn (J.L.); 2 College of Biosystems Engineering and Food Science, Zhejiang University, Hangzhou 310058, China; zjuwky@zju.edu.cn

**Keywords:** milk fatty acids, oxidative stability, dairy cows, stocking density

## Abstract

**Simple Summary:**

The ratio of unsaturated fatty acids (UFAs)/saturated fatty acids (SFAs) of milk fat is known to play an important role in regulating milk oxidation capacity in lactating dairy cows. Recently, it was found that dietary antioxidant addition plays an important role in changing UFAs/SFAs ratio of milk fat and milk oxidation capacity in dairy cows, indicating the role of oxidative stress status in regulating milk oxidation capacity. Although stocking density can affect stress level in lactating dairy cows, whether stocking density may affect milk oxidation capacity remains to be answered. In the current study, we stocked the cows with different physiological stages (mid- and late-lactating) under different stocking density (100% vs. 75%), respectively. The plasma variables, milk fatty acids compositions, and oxidative index in the milk were further evaluated to clarify if stocking density can regulate the oxidation capacity of the milk. The results showed that, in mid-lactating cows, milk produced by low stocking density animals had lower oxidation stability due to the higher UFAs levels, compared with those of high stocking density cows. Milk oxidation capacity of late-lactating dairy cows did not differ when they were blocked under different stocking density. Our results suggested that stocking density did affect milk oxidation capacity but in a physiological-stage dependent manner. Nutritional strategies should be developed to improve the milk oxidation capacity of cows managed under a low stocking density.

**Abstract:**

The objective of the current study was to investigate the effects of stocking density (SD) on the milk fatty acid profile and oxidation stability in mid- and late-lactating cows. Twenty-four multiparous mid-lactating Holstein dairy cows (milk yield = 34.5 kg/d (standard deviation 0.68), days in milk = 106 (standard deviation 2.2)) and 24 multiparous late-lactating animals (milk yield = 31.8 kg/d (standard deviation 0.98), DIM = 201 (standard deviation 3.5)) were allocated into 12 blocks based on parity, milk yield, and DIM and were randomly assigned to one of four treatments: mid-lactating cows with high SD (HSD, 100%), mid-lactating cows with low SD (LSD, 75%), late-lactating cows with HSD, and late-lactating cows with LSD. The experiment lasted for eight weeks. Lactation performance (milk yield and composition including fat, protein and lactose) was measured weekly. The milk fatty acids (FAs) profiles and oxidation stability indexes in milk were measured in the fourth day of the eighth week. Plasma variables were measured in the fourth day of fourth and eighth experimental weeks. Yield and composition (protein, fat and lactose) were not affected by SD in neither mid- nor late-lactating dairy cows. Among mid-lactating cows, LSD cows had higher contents of unsaturated FAs (total, C18:1 cis-9 and C18:2 cis-9 cis-12) and lower concentrations of saturated FAs (total, C14:0 and C16:0) in milk fat than those of the HSD animals. Moreover, LSD lowered SOD, GSH-px, and T-AOC activities and reduced the malonaldehyde content in the milk of mid-lactating cows compared with those of HSD cows. Mid-lactating cows under LSD had reduced cortisol and greater MDA contents in plasma than those of HSD cows. Our results suggested that the effect of SD on the milk FA profile and stability varied depending on lactation stages. In mid-lactating cows, although cows with LSD were less stressed, the milk they produced had lower oxidation stability due to the higher unsaturated FAs levels compared with that of HSD cows.

## 1. Introduction

Milk fat is an important dietary source of nutrients for humans, which contains about 60% saturated fatty acids (SFAs) and 30% unsaturated fatty acids (UFAs, [1,2]). The UFAs/SFAs ratio in milk fat is of great interest due to their influence on health status of consumers [3,4,5]. Previous studies suggested that dietary UFAs/SFAs can affect UFAs/SFAs ratio in the milk [6,7]. For example, compared with cows fed a diet with added palm oil, dairy cows fed diets supplemented with linseed oil and whole linseed had higher milk UFAs concentrations due to the relatively greater UFAs contents in linseed oil and whole linseed [6], indicating that ratio of UFAs/SFAs can be influenced by feeding diet with different UFAs/SFAs ratio. Moreover, Zhao et al. (2013) found that cows fed with lipid sources containing high levels of long-chain FAs and UFAs produced milk with lower antioxidant enzyme activity and higher lipid peroxidation due to the increased levels of cis-9, trans11-CLA, 18:2n-6, and 18:3n-3 in the milk fat [7], indicating that greater UFAs levels in milk fat were associated with more severe oxidation in milk. 

In addition to the UFAs/SFAs ratio in the diet, antioxidant addition can impact the proportion of SFAs and UFAs in the milk. For example, Wang et al. (2010) reported that when cows were fed diets containing lower levels of SFAs, adding antioxidants reduced the concentrations of C12:0 and C14:0 and increased the content of C18:1 cis-9 in the milk fat [8]. Similarly, when dairy cows were fed with an antioxidant mixture (containing both of selenium and vitamin E), concentrations of C10:0 and C12:0 were increased, while total UFAs levels were reduced in milk fat compared to the milk of cows fed a standard diet [9]. The information suggested that oxidative status of cows was a regulator of milk fat UFAs/SFAs ratio and milk oxidation.

Stocking density (SD) is an important index in dairy management. In most of previous studies, it is found that higher SD reduced lying and ruminating [10,11] but did not affect feed intake [12,13] of dairy cows during mid and late lactation. Interestingly, a recent study reported that a higher SD of transition dairy cows produced greater plasma cortisol concentrations [14], suggesting that cows under higher SD had greater oxidative stress. However, it is still needed to be investigated whether increasing SD can change oxidative stress status, milk fatty acids profiles and milk oxidation capacity in mid- and late-lactating dairy cows.

In China, SD rates of most dairy farm ranged from 70 to 92% (personal communication), Previous finding suggested that an average stall occupancy of only about 75% when stall SD arrived at 100%, indicating that an SD less than 100% would be more preferable by early lactating dairy cows (Wagner-storch et al., 2003, [15]). Thus, we hypothesized that cows with higher SD (100%, HSD) had reduced milk UFAs concentrations but better lipid peroxidation in milk fat than cows with lower SD (75%, LSD). Thus, the current study was conducted to investigate the effect of SD on plasma variables, milk fatty acids composition and milk oxidation stability indexes in mid- and late-lactating dairy cows. 

## 2. Materials and Methods 

### 2.1. Animals, Diets and Experimental Design

**Figure 1:**
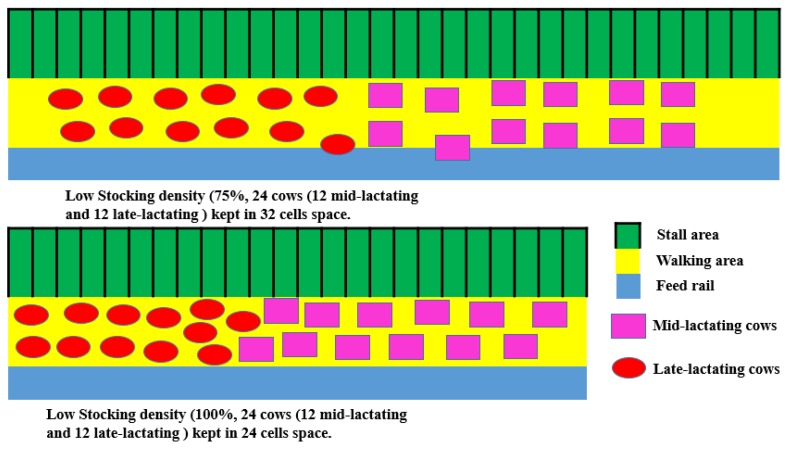
The housing design of the experiment.

All animal care and procedures were approved and conducted under the established standards of Zhejiang University (IDS1703), Hangzhou, China. Twenty-four multiparous mid-lactating Holstein dairy cows (body weight (BW) = 590 kg (standard deviation 12.3); parity = 3.21 (standard deviation 0.102); milk yield = 34.5 kg/d (standard deviation 0.68), days in milk = 106 (standard deviation 2.2)) and 24 multiparous late-lactating animals (BW = 599 kg (standard deviation 11.2); parity = 3.16 (standard deviation 0.143); milk yield = 31.8 kg/d (standard deviation 0.98), DIM = 201 (standard deviation 3.5)) were selected and allocated into 12 blocks based on parity, milk yield, and DIM and were randomly assigned to one of four treatments: mid-lactating cows with HSD, mid-lactating cows with LSD, late-lactating cows with HSD, and late-lactating cows with LSD. Mid- and late-lactating cows in the LSD condition were housed in a barn with 32 stalls and feed bins, and animals in the HSD condition were kept in a barn with 24 stalls and feed bins. In details, for low-stocking density (12 mid- and 12 late-lactating dairy cows were kept together), 32 stalls (2.4 m × 1.2 m each) were provided, with 31.2 m^2^ total feed rail (0.75 m × 41.6 m), the total walking area is 160 m^2^. Thus, for each low-stocking density cow, average 11.8 m^2^ was available. For high-stocking density (12 mid- and 12 late-lactating dairy cows were kept together), 24 stalls (2.4 m × 1.2 m each) were provided, with 23.4 m^2^ total feed rail (0.75 m × 31.2 m), the total walking area is 120 m^2^. Thus, for each high-stocking density cow, 8.9 m^2^ was available (Figure 1). Total mixed ratio (TMR) was provided to the cows daily at 0630, 1400 and 1900. Both mid and late-lactating dairy cows were fed TMR with the same diet (Table 1) according to the NRC (2001, [16]), which met the nutritional requirements of all experimental cows. Cows were housed in free stall barns and had free access to feed (fed ad-libitum, about 10% orts was left daily) and fresh water during the experimental period. The cows were milked three times daily, and the experimental period lasted for eight weeks.

### 2.2. Sample Collection and Measurement

#### 2.2.1. Feed Sample

The TMR samples were collected weekly for chemical composition analysis. The samples were dried at 65 °C for 48 h, ground through a 1-mm screen mesh using a high-speed grinder (Tecator 1093, Hoganas, Sweden) and stored in closed plastic bottles at 4 °C. After the experiment was finished, all the TMR samples were mixed together and used to determine variables following the method described in AOAC (1990, [17]) including DM content (No. 934.01), CP (No. 955.04), EE (No. 920.39), ash (No. 942.05), and ADF (method No. 973.18). The NDF was analyzed by the method described by Van Soest et al. (1991, [18]).

#### 2.2.2. Lactation Performance and Body Weight Change

The weekly milk yield for each cow was recorded on the third and fourth days of each week during the experimental period using a milk-sampling device (Waikato Milking Systems NZ Ltd., Waikato, Hamilton, New Zealand). The weekly milk samples were collected on the 4th day of each week to determine milk composition (fat, protein and lactose) using a Foss FT+ instrument (Foss-4000, Foss, Hillerød, Denmark), and milk urea nitrogen (MUN) content was measured using a modified Berthelot reaction method (ChemSpec 150 Analyzer, Bentley Instruments, Wilcox et al., 1966, [19]). On the fourth day of the eighth experimental week, a set of milk samples from individual cows was collected and stored at −20 °C. Within 24 h, the stored milk samples were used to determine FAs profile and oxidation capacity variables. Animal body weight (BW) was calculated (on day 0 and 56) by measuring body length and heart girth using the following equation: BW (kg) = heart girth^2^ (m) × body length (m) × 90 [20]. The individual BW change (BW) = final BW − initial BW

#### 2.2.3. Plasma Variables

Blood samples were collected from the coccygeal vein of individual cows using tubes containing anticoagulant (heparin lithium) 3 h after morning feeding on the 4th day of the 4th and 8th weeks. The blood samples were centrifuged for 15 min (speed 3000× *g*) to prepare plasma. The collected plasma was stored at −20 °C in the laboratory and used for variables analysis in the next day relatively to sampling day (5th day of the 4th and 8th weeks, respectively). Plasma samples were analyzed using an Auto-Analyser 7020 (Hitachi High-technologies Corporation, Tokyo, Japan) with commercial colorimetric kits (Ningbo Medical System Biotechnology Co., Ltd., Ningbo, China) to determine urea N and nonesterified fatty acids (8). The oxidative stress variables, including SOD [21], GSH-px [22], T-AOC [23], and malonaldehyde (MDA) [24] were measured with commercial kits. The cortisol concentration was measured according to the method described by Hopster et al. (1999, [25]).

#### 2.2.4. Milk FAs Determination

The frozen milk samples from each cow from the 8th week were thawed in a 4 °C refrigerator. Then, samples taken from individual cows (~10 mL) were centrifuged for 30 min at 17,800× *g* (4 °C) to separate the fat cake. Total lipid extraction from the fat cake was conducted following the protocol developed by Hara and Radin (1978, [26]), who used an n-hexane/isopropanol (3:2, vol/vol) method. After that, fatty acid methyl esters (FAME) was prepared through shaking a combination of 2.5 mL of n-hexane containing 25 mg of lipids and 0.5 mL of sodium methoxide solution (0.5 M) in methanol (5 min). Then, one gram of sodium bisulfate was added and vortexed in the vial. Finally, the vial was centrifuged for 5-min at 6000× *g*, and the supernatant containing FAME was transferred to a 2-mL vial and used to determine the FAs profile using GLC analysis [27].

#### 2.2.5. Oxidation Capacity Variables in Milk

To evaluate the radical scavenging indexes in the milk (SOD, GSH-Px, CAT, T-AOC, and MDA), milk samples from individual cows were thawed and centrifuged for 30 min at 13,000× *g* at 4 °C, and the supernatant was collected for later analysis. The measurement of SOD, GSH-px, T-AOC, and MDA were conducted with the same methods described in plasma measurement. The CAT activity in the milk was determined with an ammonium molybdate spectrophotometric method described by Goth (1991, [28]).

### 2.3. Calculation and Statistical Analysis

All data were analyzed using PROC MIXED with the STATISTICAL ANALYSIS SYSTEM software (SAS Institute, 2000, [29]). For the yield and composition of milk and variables in plasma, a randomized block design with repeated measurements was used, with week, SD, period, SD × period, block, SD × week, period × week, and SD × period × week as fixed effects and cows within treatment as the random effect, and interactions using the covariance type auto-regressive order 1 [AR (1)]. For milk FA profile and oxidation capacity variables, a completely randomized design was used, and the variation in the model with SD, period, SD × period, block. For both analyses, the results are reported as least squares means. Mean comparisons across treatments were conducted when the interaction terms of the model were significant (*p* ≤ 0.05) or tended to be significant (0.05 < *p* ≤ 0.10) by using LSMEANS and PDIFF separation of all treatments.

## 3. Results

The effects of SD on lactation performance is presented in Table 2. Throughout the whole experimental period, milk performance (milk yield, milk compositions and their production) and BW did not differ between cows under HSD and LSD in both of mid and late lactating periods (*p* > 0.05). Throughout the whole experimental periods, no effect of week × treatment (parity, SD, or SD × parity, *p* > 0.05) on lactation performance was observed.

The effects of SD on plasma variables in lactating dairy cows are presented in Table 3. In mid-lactating dairy cows, concentrations of cortisol (*p* < 0.01) and MDA (*p* = 0.02) in the plasma were greater in HSD-cows than those of LSD-animals, respectively. While other variables, including NEFA, BHBA, BUN, SOD, GSH-Px, T-AOC, and MDA, were not different between the treatments (*p* > 0.05). In late-lactating animals, no difference was observed between cows under HSD and LSD treatments (*p* > 0.05). Throughout the whole experimental periods, no effect of week × treatment (parity, SD, or SD × parity, *p* > 0.05) on plasma variables was observed.

The effects of SD on milk FAs profiles in mid- and late-lactating dairy cows are presented in Table 4. In mid-lactating animals, UFAs (*p* < 0.01) levels were lower in LSD-cows than that of HSD-animals, and SFAs levels were higher in LSD-animals compared with that of HSD-cows (*p* < 0.01). In terms of specific FAs, C14:0 (*p* = 0.02) and C16:0 (*p* = 0.02) concentrations in milk were lower in LSD-animals than that of HSD-cows. Concentrations of C18:1 cis-9 (*p* < 0.01) and C18:2 cis-9 cis-12 (*p* = 0.03) in milk were greater in LSD-animals than those of HSD-cows, respectively. In late-lactating cows, milk FAs profiles did not differ between cows under HSD and LSD treatments (*p* > 0.05).

The effects of SD on oxidation capacity indexes in milk from lactating cows are listed in Table 5. In mid-lactating cows, LSD-animals had lower SOD (*p* = 0.02), GSH-px (*p* < 0.01) and T-AOC (*p* = 0.01) activities in the milk than those of the HSD-cows. While MDA concentration in the milk was greater in LSD-cattle than that of HSD-cows (*p* < 0.01). The CAT activity in the milk was similar between the cows under HSD and LSD treatments (*p* > 0.05). In late-lactating cows, SOD, GSH-px, T-AOC, MDA and CAT did not differ between animals with HSD and LSD treatments (*p* > 0.05).

## 4. Discussion

Previous studies showed that for both mid-lactating dairy cows (DIM round 100) and late-lactating cows (DIM approximately 200), milk performance, including yield and composition, did not differ between cows with different SD [13,30,31]. Although experimental designs were different among the Pence (2005, [30]), Telezhenko et al. (2012, [31]), and Wang et al. (2016, [13]) studies and the current study, it was in agreement that low or high SD did not impact milk yield or milk compositions (protein, fat, lactose and MUN) of both mid- and late-lactating cows. In addition, previous studies also suggested that DMI was not affected in mid or late lactating cows stocked under high-density or low-density conditions [13,30,31]. Although feed intake was not measured in the current study (cows were fed ad-libitum) due to facility limitations, the similar NEFA and BHBA levels in the plasma and BW change suggested that the energy balance was not different between cows with different SD at neither the mid- nor late-lactating stages. Moreover, the similar urea nitrogen concentrations in both of plasma and milk indicated that the nitrogen conversion rate was similar between cows with HSD and LSD treatments in both mid- and late-lactating periods. Thus, similar milk production, nitrogen efficiency, and energy balance indicated that dry matter intake (DMI) between cows with different SD at either the mid- or late- lactating stages are similar, which is consistent with previous studies [13,30,31].

Similar with that of transition cows [14], when mid-lactating dairy cows were housed in a HSD condition, a higher plasma cortisol concentration was observed than that in LSD animals, indicating that in addition to transition cows, HSD elevated the oxidative stress of cows in mid-lactating stage. In relative to LSD-cows in mid-lactating period, the higher plasma MDA and cortisol concentrations in HSD-animals suggested higher levels of oxidative stress in those cows. In contrast, the different SD did not affect oxidative variables in the plasma of late-lactating dairy cows. Thus, this suggested that oxidative stress in responding to SD change can be varied, depending on lactation stages.

In mid-lactating dairy cows, the increased concentration of UFAs in the milk from LSD-cows may be attributed to the greater C18:1 cis-9 and C18:2 cis-9 cis-12 in their milk, compared with that of HSD-cows. The reduced milk SFAs in LSD-animals might be due to their relatively reduced concentrations of C14:0 and C16:0. In brief, being consistent with other studies [8,9], our study revealed that cows with less severe oxidative stress from adding antioxidants or less SD-mediated stress (the current study) had greater total UFAs and lower total SFAs concentrations in their milk. However, the change in milk UFAs and SFAs contents in different studies seemed to be a result of different FAs. For example, Wang et al. (2010, [8]) found that C18:1 cis-9 and C18:2 trans-10 cis-12 were greater and C12:0 and C14:0 were reduced in cows after addition of a commercial product containing anti-oxidants than those of cows fed a control diet [8]. While Liu et al. (2008, [9]) found that C10:0 and C16:0 concentrations in the milk were reduced in cows after they were fed with combined antioxidants (selenium and vitamin E). Moreover, although total UFAs levels were greater after dietary antioxidants were added in both studies, no change in specific UFAs were observed [8,9]. The data indicated that changing oxidative stress with different methods can affect milk UFAs and SFAs concentrations but through different types of FAs.

As a basic chemical reaction occurring in food, lipid oxidation can lead to deterioration in sensory and nutritional quality [5]. When lipids are oxidized, initial free radicals are synthesized from the UFAs, which further staring the following oxidation reaction [32]. Moreover, some products formed from lipid oxidation may affect the quality and flavor of the milk or milk products. Previous studies suggested that the oxidation capacity of milk fat can be related to antioxidant enzymes, such as SOD, GSH-Px, and T-AOC, which were reported to eliminate free radicals [33,34,35]. Our study found that HSD treatment had a positive effect on SOD, GSH-px, and T-AOC activities in milk of mid-lactating cows. Enzymatic radical scavenging systems were obviously not high enough to prevent oxidation of UFAs in the milk from LSD-cows in mid-lactating stage relative to that of HSD-animals, due to the higher milk MDA concentrations of LSD-cows. Moreover, milk with higher levels of UFAs, such as 18:2 and 18:3, in milk fat will be more easily oxidized [7,36]. In the current experimental condition, greater levels of C18:1 cis-9 and C18:2 cis-9 cis-12 in milk fat may have contributed to the more severe peroxidation in LSD-cows than that of HSD-cows in mid-lactating period. During late-lactating stage, milk oxidation capacity was not changed between HSD- and LSD-animals, which might be attributed to the similar milk FAs profiles between HSD- and LSD-cattle. The results indicated that, when mid-lactating cows were housed in an HSD environment, the milk oxidation capacity would be better, compared with animals in LSD condition. However, the effect might be limited in late lactating cows.

## 5. Conclusions

In the mid-lactating stage, LSD-cows had less oxidative stress, higher concentrations of UFAs (C18:1 cis-9 and C18:2 cis-9 cis-12), and lower SFAs levels (C14:0 and C16:0) in milk fat than those of HSD-cows. Compared with HSD-cows, the relevant antioxidant enzyme activities in milk of LSD-cows were lower, and lipid peroxidation was greater due to the higher proportions of 18:1 cis-9 and 18:2 cis-9 cis-12 in their milk fat. Also, SD change did not affect the milk FAs profiles and milk oxidation capacity in late-lactating cows. Our study suggested that although LSD reduced oxidative stress in mid lactating dairy cows, their raw milk might be more easily oxidized, which may reduce the shelf-life of the milk product.

## Figures and Tables

**Table 1:** Feed composition and ingredients of the basal diet (sample analysis = 1).

Item, % of Dry Matter	Composition, %
Alfalfa hay	13.80
Corn silage	36.86
Distillers dried grains with solubles	13.45
Corn	13.10
Soybean meal	9.33
Whole cottonseeds	3.80
Steam-flaked corn	4.10
Sugar beet pulp	3.90
Calcium bicarbonate	0.15
Salt	0.27
Limestone	0.45
Dicarbonate	0.36
Magnesium oxide	0.13
Premix ^1^	0.30
Chemical composition, % of dry matter	
Crude protein	16.8
Neutral detergent fiber	32.1
Acid detergent fiber	17.9
Ca	0.94
P	0.43
Ash	7.79
NE_L_, Mcal/kg of DM	1.68

^1^ Formulated to contain (per kilogram of DM): 150–180 g of NaCl, 250,000 IU of vitamin A, 50,000 IU of vitamin D3,1100 IU of vitamin E, 3000 mg of Zn, 17 mg of Se, 36 mg of I, 600 mg of Fe, 8 mg of Co, 630 mg of Mn and 650 mg of Cu, water ≤ 100 g.

**Table 2:** Effect of stocking density on lactation performance in mid and late-lactating dairy cows.

Items	Treatment ^1^				Standard Error of Means	*p*-Value		
	Mid		Late			SD	Stage	SD × Stage
	LSD	HSD	LSD	HSD				
Milk yield, kg								
Milk	36.3	36.1	27.9	28.5	0.38	0.66	<0.01	0.34
Fat	1.29	1.27	1.03	1.06	0.023	0.96	<0.01	0.84
Protein	1.05	1.06	0.95	0.97	0.016	0.75	<0.01	0.56
Lactose	1.80	1.82	1.41	1.45	0.021	0.46	<0.01	0.38
Milk compositions, g/100 g								
Fat	3.54	3.5	3.69	3.67	0.051	0.57	<0.01	0.91
Protein	2.89	2.93	3.43	3.36	0.040	0.75	<0.01	0.21
Lactose	4.97	4.98	5.03	5.07	0.048	0.21	0.19	0.52
MUN, mg/dL ^2^	12.9	12.7	12.8	12.9	0.30	0.86	0.61	0.46
BW ^3^ change, kg/cow	16.1	16.8	23.8	22.4	2.62	0.82	<0.01	0.54

^1^ LSD = low stocking density (LSD); HSD = high stocking density (HSD); Mid = mid-lactating dairy cows; Late = late lactating dairy cows. Weekly milk yield and compositions from individual animal (*n* = 48 for each week) were measured. ^2^ MUN = Milk urea nitrogen; ^3^ BW = body weight.

**Table 3:** Effect of stocking density on plasma variable in mid- and late-lactating dairy cows.

Items ^1^	Treatment				Standard Error of Mean	*p*-Value		
	Mid		Late			SD	Stage	SD × Stage
	LSD	HSD	LSD	HSD				
Cortisol, ng/mL	3.96 ^a^	5.24 ^b^	4.05	4.18	0.233	<0.01	0.08	0.02
NEFA, umol/L	334	373	371	364	15.2	0.29	0.39	0.13
BHBA, umol/L	490	499	505	502	9.9	0.82	0.39	0.58
BUN, mmol/L	5.37	5.39	5.43	5.52	0.091	0.58	0.30	0.66
SOD, U/mL	106	109	103	101	1.95	0.85	0.16	0.81
GSH-Px, U/mL	117	120	115	117	2.53	0.33	0.62	0.76
T-AOC, U/mL	3.62	3.55	3.65	3.58	0.088	0.85	0.86	0.38
MDA, mg/mL	173 ^a^	194 ^b^	171	180	6.5	0.03	0.18	0.04

^1^ NEFA = non-esterified fatty acids; BHBA = β-hydroxybutyric acid; BUN = blood urea nitrogen; SOD = Super oxide dismutase; GSH-px = Glutathione peroxidase; T-AOC = total antioxidant capacity; MDA = Malonaldehyde; Blood was sampled on the fourth day of the fourth and eighth weeks of individual cows (*n* = 48 for each week). ^a,b^ Means within a row with different superscripts differ (*p* < 0.05).

**Table 4:** Effect of stocking density on milk fatty acids (FA) concentrations in mid and late lactating dairy cows.

Items ^1^	Treatment				Standard Error of Mean	*p*-Value		
	Mid		Late			SD	Stage	SD × Stage
	LSD	HSD	LSD	HSD				
FA summary, g/100 g								
UFA	32.5 ^b^	29.1 ^a^	30.9	30.7	0.60	<0.01	0.91	<0.01
SFA	67.5 ^a^	70.9 ^b^	69.1	69.3	0.54	<0.01	0.92	<0.01
Selected individual FA								
C4:0	2.96	2.88	3.05	3.20	0.091	0.31	0.11	0.46
C6:0	1.94	1.77	1.95	2.09	0.105	0.26	0.13	0.54
C8:0	1.42	1.28	1.08	1.32	0.108	0.22	0.56	0.35
C10:0	3.30	3.23	3.38	3.31	0.044	0.93	0.07	0.14
C12:0	3.43	3.40	3.47	3.69	0.083	0.24	0.06	0.16
C14:0	11.5 ^a^	13.2 ^b^	12.2	11.6	0.58	0.19	0.50	0.05
C14:1, cis-9	1.03	0.97	1.05	0.95	0.049	0.11	0.94	0.64
C16:0	29.6 ^a^	31.4 ^b^	30.2	30.8	0.52	0.03	0.95	0.05
C16:1, cis-9	1.82	1.71	1.71	1.72	0.059	0.25	0.25	0.22
C18:0	9.6	10.3	8.9	10.4	0.73	0.45	0.68	0.14
C18:1, trans-9	0.54	0.47	0.48	0.58	0.061	0.76	0.69	0.22
C18:1, trans-10	0.72	0.70	0.68	0.71	0.047	0.87	0.71	0.61
C18:1, trans-11	0.88	0.94	0.90	0.96	0.046	0.91	0.73	0.21
C18:1, cis-9	20.8 ^b^	18.0 ^a^	19.6	19.2	0.51	<0.01	0.89	0.02
C18:1, cis-11	0.90	0.74	0.65	0.82	0.091	0.81	0.39	0.14
C18:2, cis-9, cis-12	3.17 ^b^	2.74 ^a^	2.71	2.84	0.16	0.86	0.40	0.04
C18:2, cis-9, cis-12, cis-15	0.24	0.29	0.32	0.28	0.022	0.62	0.29	0.30
C18:2, trans-9, trans-11	0.59	0.66	0.60	0.61	0.045	0.87	0.37	0.48
18:2, trans-10, cis-12	0.013	0.025	0.024	0.027	0.0051	0.17	0.15	0.39

^1^ UFA = unsaturated fatty acids; SFA = saturated fatty acids. Milk samples were collected on the fourth day of the eighth experimental week (*n* = 48). ^a,b^ Means within a row with different superscripts differ (*p* < 0.05).

**Table 5:** Effect of stocking density on milk oxidative variables in mid and late lactating dairy cows.

Items ^1^	Treatment				Standard Error of Mean	*p*-Value		
	Mid		Late			SD	Stage	SD × Stage
	LSD	HSD	LSD	HSD				
SOD, U/mL	4.47 ^a^	5.18 ^b^	4.74	4.62	0.207	0.17	0.51	0.04
GSH-Px, U/mL	411 ^a^	455 ^b^	435	432	8.2	0.02	0.90	<0.01
CAT, U/mL	34.8	36.1	36.9	35.4	0.84	0.36	0.87	0.13
T-AOC, U/mL	1.44 ^a^	1.69 ^b^	1.56	1.56	0.064	0.04	0.89	0.05
MDA, mg/mL	1.41 ^b^	1.11 ^a^	1.27	1.24	0.071	0.03	0.91	0.04

^1^ SOD = Super oxide dismutase; GSH-px = Glutathione peroxidase; T-AOC = total antioxidant capacity; MDA = Malonaldehyde; CAT = catalase. Milk sample used were obtained on the fourth day of the eighth experimental week (*n* = 48). ^a,b^ Means within a row with different superscripts differ (*p* < 0.05).
